# Supplementary material for: The between competition sprint profile of intercounty female gaelic football during training and match-play: An exploratory study
Source: PLoS One. 2026 Feb 2;21(2):e0340315. doi: 10.1371/journal.pone.0340315 (PMC12863504; doi:10.1371/journal.pone.0340315)
Supplement: Attachment — Submitted filename: Response to reviewers.docx [file pone.0340315.s002.docx]

**Academic editor’s review**

**Authors comments to the academic editor:**

**Response:** The authors would like to sincerely thank the academic editor for the considerable time and care dedicated to reviewing this manuscript. We appreciate the thorough, constructive, and detailed feedback provided, both in the editorial assessment and in the accompanying reviewer-style comments. Your guidance has been invaluable in helping us strengthen the clarity and practical applications of the paper. We are grateful for the balanced evaluation of the study’s potential contribution alongside the areas in need of enhancement, and we have addressed all points raised with care and precision.

Major Comments
1. Sample Size and Generalisability
o Only 14 players’ data were retained for analysis out of 23, representing a single Division 3 team. This severely limits generalisability to elite female Gaelic football as a whole. It is important to understand this when discussing your findings across the manuscript.
o The exploratory and descriptive nature is acknowledged, but the paper should emphasise that conclusions are preliminary and hypothesis-generating rather than having a significant impact, it is important to not over emphasise findings, and place them appropriately within the context of the research investigation completed.

**Response:** As authors, we agree that the exploratory nature of the study and its associated limitations need to be emphasised, especially during the discussion. We have included additional text emphasising this (L 186, 267-268, 293, 500, 697). Exploratory work ultimately allows us to begin developing better research questions, as opposed to drawing any strong conclusions. Specific hypothesis generated are now mentioned in the future research section (L 765-771). We believe the revised manuscript now highlights these important considerations.

2. Effect Sizes and Statistical Testing
o Multiple comparisons were performed without correction (alpha not adjusted). While this is acknowledged due to the exploratory design, readers should be cautioned more strongly about inflated Type I error.

**Response:** The authors thank the reviewer for this comment, it is a sharp observation and is important to point out to the readers. We have highlighted the performance of multiple comparisons without correct in the limitations section (L 702).

o At times, non-significant findings with “approaching significance” are over-interpreted. These should be discussed cautiously.

**Response:** The authors thank the reviewer for this comment. Approaching significance is no longer mentioned in the updated manuscript.

3. Training Session Context Missing
o The manuscript admits that training content was not recorded. This is a major limitation: without knowing whether training focused on technical, tactical vs conditioning drills, these are common within championship and naturally decrease the running demands of training, as there is a focus on tactical clarity and freshness leading into more important games, it is important to acknowledge this when interpreting sprint and acceleration data, without this, your elucidations are very speculative.

**Response:** We thank the editor for this comment. We have acknowledged this limitation (L 700-702), and we agree that the absence of recorded training content constrains the interpretation of sprint and acceleration data. Our aim was to generate hypotheses in an area where data on running performance across training and matches in LGF remain limited. Although training content was not objectively documented, we can note that, in line with traditional periodisation, sessions shifted from more conditioning-oriented drills during the league phase toward greater technical and tactical work during the championship. We have clarified this point in the manuscript to strengthen the interpretation of results.

4. Use of GPS Technology
o While validity of 10-Hz GPS is noted, differences in device accuracy compared to other studies are not fully considered when comparing values across sports, include as limitation or provide additional support for validity and reliability.

**Response:** We acknowledge the methodological considerations related to GPS technology and their impact on comparability across studies. This point has been clarified in the manuscript, with examples provided in L 579-580.

5. Terminology and Definitions
and the intermediate championship. More precise terminology (e.g., “inter-county intermediate-level players”) would enhance clarity.

**Response:** To date the term ‘elite’ has been used to describe any team that competes at intercounty level in Gaelic games research, but we have amended the title of the manuscript and replaced the term “elite” with “intercounty”. In the methods section, it is stated the league and championship competition that the team competed in.

Minor Language, Grammar, and Punctuation Issues
*Abstract*
• Line 32: “…with limited data on players’ sprint profiles.” Replace “with” but limited data exist on players’ sprint profiles.” for clarity.

**Response:** Amended as suggested.

• Line 33: “and provide descriptive data by positional group.” → Better phrased as “…and to provide descriptive data by positional group.”

**Response:** Amended as suggested.

• Line 35: “…across 15 games and 34 training sessions.”
Sample size is relatively small; consider flagging this as a limitation within manuscript limitations and further research section within discussion

**Response:** Added as a limitation at the end of the discussion in L 696-697.

• Line 41: “…significantly greater in league play (large effects, p < 0.05).” The phrase “significantly greater… (large effects, p < 0.05)” risks overstating exploratory data; note just large effects were observed between league and championship, given low sample size.

**Response:** Amended as suggested; “significantly” removed.

• Line 42: “…unexpected given the firmer surfaces typical of summer championship games.” Add nuance: surface conditions may not be the only explanatory factor (coaching, tactical demands, focus on freshness over fatigue during championship, importance of competition etc.) Not all the variance is explained by surface, oversimplification of your findings and has very little coaching value, what do your findings mean for the coaches on the ground.

**Response:** We agree, firmer playing surface are one variable that may explain some variation in sprint profile, but not all. Amended as suggested at the second last sentence of the abstract (L 40-42).

*Introduction*

• Line 47: “…dynamic requiring players…” Insert comma: “…dynamic, requiring players…”

**Response:** This sentence has been amended with improved phrasing.

• Line 55-59: “….Athlete monitoring enables the sports science practitioner to determine athletes’ internal and external responses to training loads and match-play (7,8). These data can allow practitioners to prepare optimally for competition by planning and adjusting training loads to reduce the risk of non-functional over-reaching which may result in an increased risk of illness or injury (Kelly et al., 2022; O’Connor et 58 al., 2021; (7–9)….” As your research is focused on external load and locomotor patterns of the game, there is no need to reference internal loading, I would suggest a restructure of this paragraph

**Response:** Thank you for your observation; while internal load monitoring can play an important role in monitoring an athlete’s response to training, it is not the subject of this manuscript and thus should be removed. The beginning of the second introduction paragraph is amended to reflect this (L 73-75).

• Line 57: “…non-functional over-reaching which may result…” Hyphenation is not required: “nonfunctional overreaching, which may result…”

**Response:** Amended as suggested (L 75).

• Lines 58–60: Delete Kelly et al., 2022; O’Connor et al., 2021 and keep reference number (7–9).”

**Response:** Amended and removed

• Line 63: “…over 95% of the research in Gaelic games has been conducted in the male population (16).” Suggest rephrasing: “within male cohorts”

**Response:** Amended as suggested (L 81).

• Lines 66–67: Repetition of “consequently” in two consecutive sentences, need to improve the flow of your sentence construction here.

**Response:** The authors agree that repetition should be removed. Further, the flow of the sentences has been amended and improved (L 82-86).

• Line 99: “with a secondary aim was to provide…” Grammar issue here: “with a secondary aim to provide”

**Response:** Amended as suggested (L181-182).

*Methods*

• Line 134: “…written consent was obtained prior to their participation..” Double period remove one.

**Response:** Double period removed

• Line 138-139: “….The validity of these devices in measuring total distance and peak speed during shuttle run tasks compared to a video camera has been shown to be acceptable…..” – Provide the validity and reliability of the specific metrics of Accel, Decel and sprint demarcations used within your investigation, if these can not be found provide data from within your research group on the validity and reliability of these data.

**Response:** We thank the reviewer for raising this point. To the best of our knowledge, published validity and reliability data specific to the acceleration, deceleration, and sprint thresholds used in this study are not currently available in the literature, nor does our research group possess unpublished data addressing these metrics. We have therefore retained the broader, device-level validation statement.

• Line 145: “……players were familiarised with the devices by wearing them during training sessions and challenge games three weeks…..” Revise to “for three weeks”

**Response:** “For” added (L 229).

• Line 156: “…..For further detail, please refer to (cross-156 sectional ref)….” ….Include the reference here !

**Response:** Amended as suggested

• Line 158–159: “…this new value became their updated peak speed. (29).” Extra period before citation, remove.

**Response:** Period removed

• Line 165: “…and the highest percentage of a peak speed player’s achieved.” This is very clunky phrasing; suggest you revise to “…and the highest percentage of each player’s peak speed achieved.”

**Response:** Thank you making this suggestion, it has been amended accordingly (L 249-250).

• Line 169: “……relative speed thresholds have been used in hurling (12,30,31). The minimal effort of duration for a 168 sprint was ≥ 1.0 s 23/09/2025 06:25:00….”…..Remove the date here which appears to be a inserted comment from a fellow authorship member

**Response:** Thank you for this observation. Amended as suggested.

*Results*

**Author comment:** The authors would like to state that the statistical analysis was originally conducted in SPSS. However, to enhance the reproducibility and transparency of our work, the statistical analysis has been replicated identically in R Studio. The code for this analysis is included in the supplementary files. It is important to note that SPSS and R Studio round numbers slightly differently, and although the results are the same, some figures in tables 1 and 2 have been changed in accordance with these updated numbers. There was one decimal place error for the ES for accelerations in games which has been corrected and highlighted. All other data presented have been checked comprehensively and cross-referenced throughout the manuscript.

• Line 197-210: Include the ES number, the inference and then the P-Value, this provides clarity to all your findings. I also feel you should provide an introduction to the overall results in mean and SD and positional differences and then get into the differences between competitions. I think you need to be careful with the use of statistically significant, given small sample sizes.

**Response:** We appreciate the reviewer’s suggestions regarding presentation of the results.

We have amended the text of the results section as suggested (L 296-314). With respect to the use of statistical significance, we agree that caution is warranted given the modest sample size, and this point is emphasised throughout the discussion to ensure appropriate interpretation of the findings.

*Discussion*
• “The lack of significance in combination with the low sample size constrain inferences…” → Should be “constrains inferences…”

**Response:** This line has subsequently been removed.

• “None of these factors have yet to be investigated…” Better phrased as “…have been investigated…”

**Response:** Amended as suggested (L 565-566).

• “Since sprinting is a common cause of injury in female collegiate athletes and hamstring injuries account for 22% of injuries in collegiate female Gaelic football…” This is a long sentence and could be split for readability.

**Response:** This sentence has been amended and split into two for enhanced readability. Thank you for this suggestion (L 675).

• The authors need to add an appropriate limitations and future research section at the end of the discussion

**Response:** Thank you for this suggestion. We agree both sections are important to include and have been added after the main discussion section (L 695 and 757).

• An overall comment would be that the authors need to do more than state the findings, answer always for each paragraph, what do our findings mean for players and coaches of the sport, how will the data aid training construction and within match-play performance decisions from coaching and performance staff

**Response:** Thank you for this valuable suggestion. We agree that clearer practical implications strengthen the relevance of the findings for coaches and players. Accordingly, paragraphs 2, 4, and 6 of the discussion have been revised to more explicitly articulate actionable takeaways and how the results may inform training design and match-play decision-making.

*References*
• Inconsistent formatting of references throughout the paper, please complete review in line with journal requirements.

**Response:** Many thanks for this observation. All references have been checked and amended in line with the journal requirements.

• Inconsistent formatting for references and names and punctuation (e.g., some include full stops after abbreviations, others do not).

**Response:** Thank you for your comment. All references are now in accordance with the journal requirements.

• Ensure all cross-referenced “(cross-sectional ref)” and “(survey ref)” placeholders are replaced with complete citations.

**Response:** Thank you for your diligence and attention-to-detail here. All of the mentioned “(refs)” have been addressed throughout the manuscript and replaced appropriately.

*Tables and Figures*
Table 1
• Inconsistent p-value spacing (p <0.05 vs p < 0.05).

**Response:** Amended as suggested throughout the manuscript. Now consistently p < 0.05.

• Missing ± SD for specific metrics that need to be added

**Response:** Thank you for this comment. All the relevant ± SD are included where those data were normally distributed. Non-normally distributed data are inputted are medians with the IQR. The table caption has been amended to reflect and highlight this important distinction (L 335).

• Suggest highlighting in Bold the effect size that met the G-Power threshold for 80% Power

**Response:** Amended as suggested (L 283).

• Abbreviation key (PS = Peak Speed) should be moved to the table caption for clarity.

**Response:** Thank you for this suggestion. Abbreviation keys are placed at the bottom of the table to ensure consistency across tables.

Table 2
• Missing ± SD for specific metrics that need to be added

**Response:** Thank you for this comment. Considering the low sample included for the positional data, we have adjusted all of Table 2 to be median and IQR to have a fairer reflection of the central tendency and variability of the data. This is stated in L 309-311.

Figure 1
• Suggest making bar whisker plots and keep the individual bee swam at the back of the plot, allows for more understanding for wider sports science community.

**Response:** Thank you for your comment. This plot has been updated the include the mean and standard deviation. To comply with journal guidelines, it is not currently included in the manuscript.

• Axis labels not shown in text snippet — ensure units (e.g., “Number of sprints [n]”) are clearly labelled.

**Response:** Amended as suggested (L 462).

• Caption could better explain the plot used and how data are presented.

**Response:** Thank you for this observation. The caption has been updated to elucidate the data presented. L 462-464.

Figure 2
• This is an infographic, and while it is cool, it needs more scientific rigor applied, I suggest adding mean and SD to the figure and rounding the %Peak Speed to a round figure with SD
• Caption: “ indicates a median value”* Asterisk notation unclear; should specify “ indicates statistically significant difference (p < 0.05)”* if that is the case.
• Abbreviation (PS = Peak Speed) repeated but should be defined once.

**Response:** Thank you for this comment. In response, we have standardised sticking to mean ± SD as the median IQR is similar. Clip art has been removed.

Editorial Recommendation
Major Revisions but can be published with PLOSone post edits and reviewer revisions are completed.
The study has merit and novelty but requires:
• Stronger acknowledgment of limitations in sample size, training context, and device comparisons.
• Careful moderation of conclusions to reflect exploratory rather than confirmatory findings.
• Polishing of grammar, punctuation, and reference formatting for increased readability.

**Response:** We thank the editor for the clear guidance and for recognising the merit and novelty of the study. The revisions requested have been fully addressed. Specifically, limitations relating to sample size, training context, and device comparability have been clarified; conclusions have been moderated to reflect the exploratory nature of the work; and grammar, punctuation, and reference formatting have been carefully refined throughout. We appreciate the constructive feedback and believe these revisions have strengthened the manuscript considerably.

**5. Review Comments to the Author**

**Reviewer #1:** Thank you for the opportunity to review this paper. This is a potentially interesting paper. This area of female sports is under researched and as a result, this information could add the field. However, there are major revisions needed as the paper is tangential in places and doesn’t critically analyze the results through the discussion. The submission seems incomplete; there are errors throughout which should have been corrected pre-submission.

General feedback:
Please complete a through proof-read before re-submission. Improve writing clarity — several grammatical errors and awkward phrases reduce readability. The paper could have potential value and could fits the journal’s scope, but significant methodological and reporting issues must be addressed. The authors should refine their introduction discussion before the manuscript can be reconsidered. Your findings need to be expanded in line with best sports science practices in elite sports. There is an opportunity here for you to provided recommendations for coaches/sports scientists to replicate the demands of the game in training to ensure the players are best prepared for competition. Check reference style to ensure compliance with journal guidelines.

**Response:** We thank the reviewer for their thorough evaluation of our manuscript and for highlighting several important areas for improvement. We appreciate the recognition of the potential value and novelty of this work within the context of the limited research available in female Gaelic games. In response to the points raised, the manuscript has undergone considerable revision to enhance clarity, structure, and rigour.

A full proof-reading has been completed, with particular attention given to grammar, phrasing, and readability to ensure a more concise and coherent narrative throughout. Methodological descriptions have been clarified where required, and reporting has been standardised in accordance with journal guidelines. The Introduction and Discussion sections have been refined, with additional contextualisation, improved linkage between findings and existing literature, and the integration of more explicit practical implications relevant to coaches, practitioners, and performance staff. Furthermore, reference formatting and adherence to journal style have been comprehensively checked and corrected.

We believe these revisions substantially strengthen the manuscript and address the issues identified. We are grateful for the reviewer’s constructive input, which has directly contributed to improving the overall quality and applied value of this study.

Specific feedback:
Abstract:
Line 34: Why only 14 players?
**Response:** Thank you for your observation and comment. As stated in the methods section, due to the paired design and insufficient data for both league and championship training and games, data for fourteen players only were retained for analysis. Numerous players dropped out of the panel throughout the year, meaning those that played numerous league games and left for the championship could not be included in the analysis (L 215-216).

Line 40: Should specific effect size be reported here (p < 0.05, ES = 1.2)? p should also be in italics throughout the document. Please amend throughout the document.
**Response:** P values italicised accordingly throughout the manuscript and ES have been added into the abstract as a range.

Line 42-43: Sentence need to be re-written.
**Response:** Thank you for your comment. This sentence has been re-written.

Line 43: There is always further research needed, could more specific recommendation be provided here based on what your study found?
**Response:** Thank you for your comment. The final line of the abstract now provides a more concrete future research direction in LGF (L 42-44).

*Introduction:*
Needs to be proof-read and re-formatted throughout the section.
**Response:** Thank you for your comment here, the authors have proof-read and re-formatted the manuscript.

Line 47-49: I get what you and trying to say here, re-write and re-phrase.
**Response:** Phrasing has been improved for this second sentence of the introduction.

Line 47-54: I would suggest a little more context around the unique principles of play, and the unique formatting and structure of the NL and AIC in relation to LGF. Journal will have international readership so a greater outline of the game in needed here.
**Response:** Thank you for your comment. We have added sentences to the first paragraph in the introduction and provided more details about the sport to contextualise the game for the international audience (L 57-72).

Line 52: ‘suggested’ instead of ‘purported’, doesn’t read well.
**Response:** Amended as suggested

Line 52-54: references at the end a sentence (5, 6).
**Response:** Amended as suggested.

Egan et al., is from intercounty hurling research. I would suggested to use read the following papers which looked at some of the contextual demands in male GF:
Mangan, S., Malone, S., Ryan, M., McGahan, J., O’Neill, C., Burns, C., Warne, J., Martin, D & Collins, K. (2017). The influence of match outcome on running performance in elite Gaelic football. Science in Medicine & Football 1, 272-279.
Mangan, S., Ryan, M., Shovlin, A., McGahan, J., Malone, S., O'Neill, C., Burns, C., & Collins, K. (2019). Seasonal changes in Gaelic football match-play running performance. Journal of Strength & Conditioning Research 33, 1685-1691.
McGahan, J., Mangan, S., Collins, K., Burns, C., Gabbett, T., & O’Neill, C. (2021). Match-play running demands and technical performance among elite Gaelic footballers: Does divisional status count? Journal of Strength & Conditioning Research 24, 169-175.
Additionally, these can support/refute your findings in the discussion.
**Response:** Thank you for suggesting these papers for inclusion in both the Introduction and Discussion. The reference by Mangan et al. (2019) has been added to the end of the Introduction. The authors acknowledge that the study by Egan et al. was conducted in hurling; however, it is the only paper, to our knowledge, that addresses the perceived importance of competition. Although the Walsh study is not directly related to Gaelic games, it was included as it highlights the variation in rainfall across different months in Ireland.

Line 55: remove ‘the’, add sports science practitioners.
**Response:** Thank you for your comment. It now reads “Athlete monitoring allows sports scientists to quantify external training loads…” (L 73-74).

Line 56: This data….. delete These.
**Response:** Thank you for your comment. The phrase “These data” is grammatically correct here as “data” is the plural of ‘datum'.

Line 58: Delete Kelly et al., 2022; O’Connor et al., 2021; (7–9). Ensure in-text referencing is consistent and in line with journal recommended referencing style.
**Response:** In-text referencing has been standardised throughout.
Has this paper been submitted elsewhere?
**Response:** This paper was completed as part of a doctoral thesis, thus a small number of references have not yet been updated by reference management software. The authors confirm that this manuscript has not been published or simultaneously submitted for publication elsewhere.

Lines 61-63: Change this statement to the following:
Previous research conducted in Gaelic games using GPS technology has focused on many elements of training and competition including match-play demands, the influence of different constraints on small-sided game outputs, and the influence of training workload on injury risk (2,11–15).
**Response:** Thank you for your comment. As suggested, the word “facets” has been replaced by “elements”, and “parameters” by “constraints” (L 78-79).

Line 65: This is true but there is specific female GAA research to support your statement here. References 16 & 22.
**Response:** This has been amended accordingly (L 87).

Lines 66-68, Consequently, used twice in four sentences.
**Response:** This has been amended accordingly (L 83-87). Thank you.

Lines 70-71: Re-write, suggested:
There is currently a dearth of research conducted in intercounty female Gaelic football….
**Response:** Thank you for your suggestion.

Line 71: et al., should be in italics, with the inclusion of a comma.
**Response:** Thank you; amended accordingly.

Line 71-74 during competitive games? This is mis-leading as the research was conducted over two seasons with no differentiation between NL or AIC.
**Response:** Thank you for your comment. This sentence (now L 88-91) serves to highlight that Malone et al 2023 is the only work completed on intercounty LGF running performance, the study’s data are comprised of challenge, league, and championship games.

Lines 76-78: Please re-write and re-structure.
**Response:** Thank you for your suggestion.

Line 81: Would the Ladies GF recommendations paper be a more appropriate reference here, to compare apples with apples
**Response:** Thank you for your suggestion. References here updated (L 158).

Line 82: Reference 12 = hurling research. Very different physical, technical & tactical demands in comparison to GF. I would recommend making comparisons to LGF as much as possible and then comparison it to the male version of the game.
**Response:** Thank you for this suggestion. We acknowledge that hurling and Gaelic football differ in their physical, technical, and tactical demands. However, there is currently limited literature available in ladies’ Gaelic football, and hurling research provides the most comparable data in terms of certain aspects of play, such as defensive pursuits and opponent engagement (e.g., tackling and blocking) (L 160-165).

Please amend/adjust throughout the document.
Line 86-87: Good example of GF comparison here.
Line 94-96: In line with previous comments, are there similarities in Gaelic football.
Line 99: Ladies or female Gaelic football, keep consistent throughout the document, intercounty would be have to specified here also.
Lines 99-102: Does the journal require a hypothesis statement? Editor can advise.

**Response:** Thank you once again for your observation and diligence. References have been updated to be as relevant to the sport as possible throughout while female Gaelic football is used exclusively in the document. According to the journal’s guidelines, a formal hypothesis statement is not explicitly required in every manuscript. Further, for exploratory research, a formal hypothesis statement is generally not required as this research allows for the identification patterns/variables that may inform future research directions.

*Methodology*
This section is generally well written.
Experimental Procedures
Line 137 – The pro series has been validated as the team series yet? If not, needs to be included in the limitations section.
**Response:** Thank you for your comment. Based on the current literature, there has yet to be a study validating the team series. The primary difference between the ‘pro’ and ‘team’ series is the software used to calculate the metrics. As of the last time we checked, the firmware are the same.

Line 156-157 – ‘please refer to (cross sectional ref) as identical methods were used.’ What is this referring to?
**Response:** Thank you for your observation and comment. This reference has been amended and the appropriate reference has been inputted (L 241).

Line 157-158 – Please refer to the following papers for guidance on the utilization of relative & absolute speed thresholds.
Could be of use in the discussion section:
Gualtieri, A., Rampinini, E., Dello Iacono, A., & Beato, M. (2023). High-speed running and sprinting in professional adult soccer: Current thresholds definition, match demands and training strategies. A systematic review. Frontiers of Sports Activity and Living 5, doi: 10.3389/fspor.2023.1116293.
Park, L.A.F., Scott, D., & Lovell, R. (2019). Velocity zone classification in elite women’s football: where do we draw the lines? Science & Medicine in Football 3, 21-28.
**Response:** Thank you for drawing attention to these papers on the use of relative and absolute speed thresholds. We acknowledge that Gualtieri et al. (2023) provide valuable guidance on relative speed thresholds in female soccer, including the use of 80% and 90% of individual peak speed. In the present study, we applied thresholds consistent with Gaelic games research while aligning with this relative approach to ensure methodological coherence with sport-specific speed profiles. Accordingly, we have cited Gualtieri et al. to support our use of relative thresholds (L248–251).
Line 166-168 – are there any Gaelic Football relative speed thresholds available.
**Response:** Thank you for this comment. We have added a reference for absolute speed thresholds in ladies’ Gaelic football. To the best of our knowledge, relative speed thresholds have not yet been reported for Gaelic football, however, we have used a reference for relative speed zones used in hurling (L 251).

Lines 166-168 – slight re-structuring and split sentence into two:
The absolute speed, acceleration and deceleration thresholds have been used previously in intercounty camogie and international female field hockey. Additionally, the relative speed thresholds have been used in intercounty hurling (12,30,31).
**Response:** This has been amended accordingly. Thank you (L 239-242).
Did this paper not use similar velocity thresholds?
O’Grady M, Young D, Collins K, Keane J, Malone S, Coratella G. An Investigation of the Sprint Performance of Senior Elite Camogie Players during Competitive Play. Sport Sci Health. 2022 Jan 16.
**Response:** Correct; this paper is cited. Thank you.

Line 169- 23/09/2025 06:25:00, please delete.
**Response:** This has been amended accordingly. Thank you.

Statistical Analysis
Well written, clear and transparent, repeatable.
Results

**Author comment:** The authors would like to state that the statistical analysis was originally conducted in SPSS. However, to enhance the reproducibility and transparency of our work, the statistical analysis has been replicated identically in R Studio. The code for this analysis is included in the supplementary files. It is important to note that SPSS and R Studio round numbers slightly differently, and although the results are the same, some figures in tables 1 and 2 have been changed in accordance with these updated numbers. There was one decimal place error for the ES for accelerations in games which has been corrected and highlighted. All other data presented have been checked comprehensively and cross-referenced throughout the manuscript.

Line 200 – p value first, ES as in abstract?
Young D, Malone S, Collins K, Mourot L, Beato M, Coratella G (2019) Metabolic power in hurling with respect to position and halves of match-play. PLoS ONE 14(12): e0225947. https:// [doi.org/10.1371/journal.pone.0225947](http://doi.org/10.1371/journal.pone.0225947).
This paper may be of use for structure and formatting purposes.
**Response:** Thank you for providing us with this paper as an example for formatting and referencing, and one that is relevant to this research area.

The result section is very brief,
The descriptive statistics and effect sizes for training and games between league and championship appears to be missing?
**Response:** We thank the reviewer for this comment. We are not entirely certain about the additional information being requested. The descriptive statistics, p-values, and effect sizes for both training and game data across the league and championship phases are presented in Table 1. If further clarification is desired, we would be happy to address them upon further guidance.

Line 212 - In table 1, the championship total sprint distance is 337 ± 339, is the SD correct here?
**Response:** We thank the reviewer for this observation. This was a mistake and has been since corrected to 337 ± 186 m.

Lines 220 & 224 – Good visualization of the data here.
**Response:** Many thanks for these observations.

Discussion
General Feedback
The discussion should better integrate findings with existing literature. Currently, it reads more like a summary than a critical evaluation. Clearly distinguish between statistically significant results and practical applications for intercounty LGF players.
Can recommendations be made regarding the minimal effective dosage of specific physical qualities in line with best practice with other team sports?
Just a suggestion possible utilization of Tactical Periodization to marry physical, technical and tactical as per recommend in Gaelic Football:
Timmons, K., Collins, K., & Mangan, S. (2025). The science of Gaelic football match-play and recommendations for future research directions: a narrative review. Sport Sciences for Health, 1-17.
Mangan, S., Collins, K., Burns, C., & O’Neill, C. (2021). A tactical periodisation model for Gaelic football. International Journal of Sports Science & Coaching, 17(1), 208-219. <https://doi.org/10.1177/17479541211016269>.
**Response:** The authors would once again like to thank the reviewer for their observations and comments throughout the review. In line with your recommendations and those of the academic editor, more tangible practical recommendations have been provided in several of the paragraphs throughout the discussion section.

Please check the correct reporting of ES in the discussion as per journal guidelines.
**Response:** Thank you for your observation. The manuscript and reported values are reported and formatted in line with the journal guidelines.

Line 230- intercounty, please keep terminology consistent throughout the document. **Response:** Thank you for your comment. Inter-county is the term now used exclusively throughout the manuscript.

Line 230- Furthermore **Response:** Thank you for your comment. This has been amended accordingly.

Line 232 – performed rather than recorded? **Response:** Thank you for your comment. This has been amended accordingly (L497).

Line 235-238 - Not sure this is appropriately placed here.
Line 252 – Research conducted in elite female soccer….(35). Research at the end of sentence to enhance flow. **Response:** Thank you for your comment. This sentence’s syntax has been now improved.

Line 259-261 – very repetitive here, see feedback regarding best practices in other sports and it can be implements in LGF. **Response:** Thank you for the suggestion. The end of this paragraph has been amended. L 525-545.

Line 265 – Shorter sprint burst, colloquial, consider the international readership of the journal.
**Response:** Thank you for this observation. This has been amended accordingly. L550-556

Line 267 – first mention of solo, again reader may be unaware of what this specific skill is, please outline in introduction.

**Response:** Thank you for this observation. Solo is now outlined in the opening paragraph of the introduction (L 65).

Line 268-269 - MSS should still be exposed their MSS (vaccine) as you have previously eluded to in the introduction.
**Response:** Thank you for your observation. We have eluded to MSS exposure as having a protective effect in the introduction (L 166-167) and discussion (L 527 and 545).

Line 270 (MSS, ref) **Response:** Thank you for this observation. This has been amended accordingly and the correct reference has been inputted (L 563).

Line 270-271 – Poor sentence, re-write. **Response:** Thank you for your comment. This sentence has been rephrased.

Lines 277-279 – These actions are also linked to gal scoring opportunities in other sports.
**Response:** Thank you for your observation. This sentence has been removed.

Line 282- incomplete reference
**Response:** Thank you for noticing. This sentence has subsequently been removed.

**Response:** The correct reference has been inputted. Thank you for this observation.
Line 292- delete (cross-sectional ref) **Response:** Thank you for this observation. This has been removed.

Line 294 intercounty
**Response:** Amended accordingly (L 689).

Lines 295-302:
This may be of use to see recommendations, to help guide practice and link to your findings

Duggan JD, Byrne PJ, Malone S, Cooper S-M, Moody J. High-Intensity Accelerations and Decelerations During Intercounty Camogie Match Play. Sports Health: A Multidisciplinary Approach. 2024;17(1):66-79. doi:10.1177/19417381241276016.
Line 303 – Reference (survey ref) **Response:** Thank you; these sentences have been modified to make a stronger take-home message for practitioners (L 614-625).

Line 303 educate the players?? How about the coaches?
**Response:** Thank you for this comment. This point has been removed.

Line 331-332 Game specific training?
**Response:** This point serves to highlight the disparity between training running performance and match-play. This paragraph has been amended to provide more tangible recommendations to coaches and practitioners (L 667-680).

Line 337-338 This seems just like a throw away statement, delete. **Response:** Thank you for this comment; this sentence has been removed.

Lines 340-342 – This is concerning, did this not have a impact on data collection and analysis?
**Response:** Thank you for your comment. Reduced training adherence primarily affected the sample size available for inclusion in this study but did not compromise the accuracy or integrity of data collection and analysis.
